# Age Differences in Pertussis Epidemics, New South Wales, Australia, 2015 and 2024

**DOI:** 10.3201/eid3210.260332

**Published:** 2026-10

**Authors:** Ella F. Madden, Sally L. Ellis, Andrea Parisi, Christine E. Selvey, Janaki Amin

**Affiliations:** New South Wales Ministry of Health, St. Leonards, New South Wales, Australia (E.F. Madden, S.L. Ellis, A. Parisi, C.E. Selvey, J. Amin); University of Sydney, Sydney, New South Wales, Australia (A. Parisi); Australian National University, Canberra, Australian Capital Territory, Australia (A. Parisi); Macquarie University, Sydney (J. Amin); University of New South Wales, Kensington, New South Wales, Australia (J. Amin).

**Keywords:** pertussis, *Bordetella pertussis*, bacteria, bacterial infection, respiratory infections, whooping cough, vaccination, immunity, waning immunity, epidemic, adolescent, Australia

## Abstract

We compared data from the 2015 and 2024 pertussis epidemics in New South Wales, Australia. In 2024, we found twice as many notifications and a shift in distribution to older children. COVID-19 interventions and lack of circulation of pertussis likely contributed to an age cohort immunity gap and increased infections.

Pertussis (whooping cough) is a highly contagious respiratory disease caused by *Bordetella pertussis* bacteria. Although infants <6 months of age are at greatest risk for severe disease and death (*1*), infections occur across all age groups. Immunity from both immunization and infection wanes over time. Consequently, pertussis remains endemic in New South Wales (NSW), Australia, and causes epidemics every 3–4 years (*2**,**3*).

Pertussis vaccines are funded in Australia under the national immunization program. Since 2009, pertussis vaccines are administered at 6 weeks, 4 months, 6 months, 18 months, and 4 years of age in NSW; vaccination during pregnancy has been funded since 2015. A booster dose is given at age 12–13 through the NSW school vaccination program (*4*). The COVID-19 pandemic disrupted infection cycles, likely because of widespread nonpharmaceutical interventions that have reduced transmission of multiple respiratory pathogens (*5*). From 2020–2023, NSW reported historically low pertussis activity, with <600 notifications annually (*3*). We describe age- and sex-specific patterns across 2 epidemics in NSW, in 2015 and 2024, and discuss the potential results of pandemic-related interventions and waning immunity as likely contributors to observed pattern changes. 

We calculated age-specific rates by using annual NSW population estimates (*6*). Confirmed pertussis cases (*7*) are reported to the NSW notifiable conditions information management system, a statutory public health register of infectious diseases legally notifiable to NSW Health (*8*). Our review of those data reveals NSW experienced a resurgence in 2024, recording the highest annual total since pertussis became notifiable in 1991. After 1991, the most recent NSW epidemic occurred in 2015. During 2015–2024, a total of 68,842 confirmed pertussis cases were reported in NSW, including 12,007 in 2015 and 25,695 in 2024. Compared with 2015, notifications in 2024 more than doubled, and the age distribution shifted toward older children, with the greatest proportion of notifications in 10–14-year-olds. The notification rate in 2024 (314.7/100,000 population) was almost double the rate of 2015 (157.7/100,000 population) (Table). The ratio of male to female notifications in 2015 was 0.88; in 2024, the ratio of male to female notifications was 1.08.

**Table:** Demographic characteristics of pertussis cases notified in New South Wales, Australia, 2015 and 2024*

Characteristic	Notifications			Rate, cases/100,000 population†		IRR, 2024 vs. 2015 (95% CI)
	2015	2024		2015	2024	
Age group						
<1 y	336 (2.80)	503 (1.96)		342.47	505.03	1.47 (1.28–1.69)
0–5 mo	171 (1.42)	232 (0.90)		348.58	516.33	1.48 (1.22–1.8)
6–11 mo	165 (1.37)	271 (1.05)		336.35	603.13	1.79 (1.48–2.18)
1–4 y	1,477 (12.30)	1,641 (6.39)		372.04	431.54	1.16 (1.08–1.24)
5–9 y	3,020 (25.15)	5,964 (23.21)		617.39	1,187.25	1.92 (1.84–2.01)
10–14 y	2,666 (22.20)	9,762 (37.99)		594.18	1,904.40	3.21 (3.07–3.35)
15–24 y	981 (8.17)	2,738 (10.66)		99.50	275.72	2.77 (2.58–2.98)
25–34 y	645 (5.37)	859 (3.34)		57.77	73.85	1.28 (1.15–1.42)
35–44 y	905 (7.54)	1,185 (4.61)		87.96	105.64	1.2 (1.1–1.31)
45–54 y	853 (7.10)	1,332 (5.18)		86.51	131.25	1.52 (1.39–1.65)
>55 y	1,124 (9.36)	1,711 (6.66)		54.41	71.95	1.32 (1.23–1.43)
Sex						
M	5,607 (46.70)	12,883 (50.14)		148.47	317.32	2.14 (2.07–2.21)
F	6,386 (53.19)	12,801 (49.82)		166.32	311.78	1.87 (1.82–1.93)
Unknown/other	13 (0.11)	10 (0.04)		NA	NA	NA
Median age, y (IQR)	11 (7–33)	11 (9–17)		NA	NA	NA
Total cases	12,007 (100.0)	25,695 (100.0)		157.65	314.68	2 (1.95–2.04)

*Values are no. (%) cases except as indicated. IRR, incidence rate ratio. NA, not applicable. †As no population was available for the groups 0–5 mo and 6–11 mo, the total population estimates for people <1 years of age in 2015 and 2024 respectively was halved. Those populations were then used for rate calculations.

In 2015, the highest proportion of cases occurred in children 5–9 years of age (25.2%), followed by those 10–14 years of age (22.2%). By contrast, children 10–14 years of age dominated the 2024 epidemic caseload (38.0%), followed by those 5–9 years of age (23.2%). The percentage of cases in children <5 years of age declined from 15.1% in 2015 to 8.3% in 2024 and in children <6 months of age from 1.4% in 2015 to 0.9% in 2024. Conversely, the notification rate for children 10–14 years of age more than tripled, from 594.2 cases/100,000 population in 2015 to 1,904.4 cases/100,000 population in 2024 (incidence rate ratio [IRR] 3.21 [95% CI 3.07–3.35]) (Table). That increase was not observed to the same extent in other age groups, particularly those >25 years of age. The median age at notification remained consistent at 11 years in both epidemics. However, the interquartile range for age narrowed substantially, from 7–33 in 2015 to 9–17 in 2024 (Table), reflecting a more concentrated distribution of cases among school-age children in 2024.

The COVID-19 pandemic likely changed health-seeking and testing behaviors, resulting in a higher proportion of persons with laboratory-confirmed pertussis. That behavior change might have contributed to higher notifications in 2024, but it is unlikely to explain the pronounced shift in age distribution. Several factors possibly contributed. The near absence of circulating pertussis during the pandemic could have disrupted natural immune boosting from exposure to *Bordetella pertussis*. That disruption probably had the greatest effect on children approaching adolescence, as reflected in the age-specific rates (Figure). In 2024, the risk of infection increased with each year of age among school-age children, particularly in those 8–12 years of age, who were several years past their last scheduled vaccine dose and therefore more susceptible to illness because of waning immunity. That immunity gap likely enabled rapid transmission through school populations. In contrast, the relative reduction in the age-specific rates from age 13 years onward, seen in both epidemics but more noticeably in 2024, signals the effect of the adolescent booster and its importance in maintaining immunity (Figure). The NSW pertussis resurgence in 2024 aligns with international observations after the easing of COVID-19 restrictions, with increased incidence among older children reported by several countries (*9*). Those parallels point to global immunity gaps attributable to reduced pathogen circulation during the pandemic.

**Figure:**
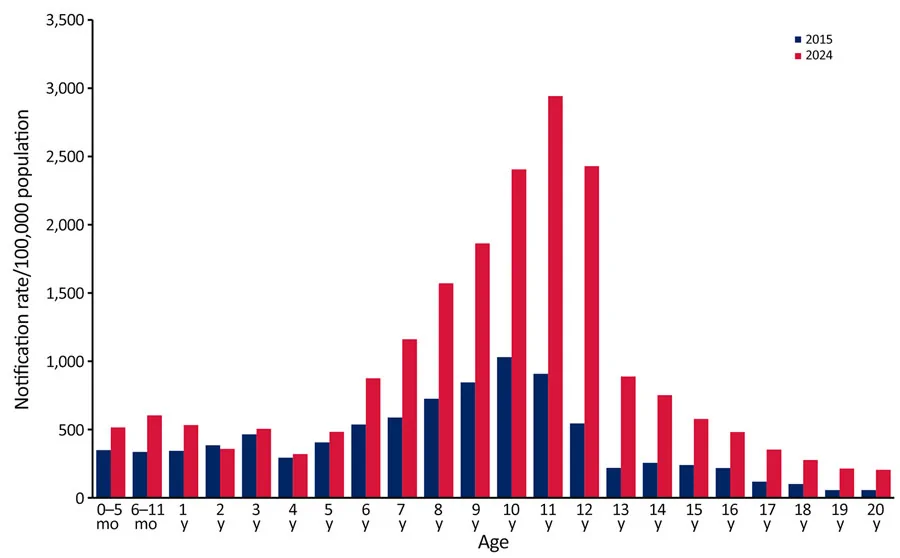
Age-specific pertussis notification rates per 100,000 population for persons 0–20 years of age, by year notified, New South Wales, Australia, 2015 and 2024. Because no population data were available for children 0–5 months and 6–11 months, the total population estimates for children <1 year of age in 2015 and 2024 was halved. These populations were used for the rate calculations.

In conclusion, the 2024 NSW pertussis epidemic differed substantially from the 2015 epidemic in both magnitude and age profile. Waning immunity, exacerbated by reduced natural boosting during the COVID-19 pandemic, likely contributed to the increased effects, particularly in children 8–12 years of age. Future research should explore vaccine effectiveness during extended inter-epidemic periods and the role of adolescent boosters to mitigate transmission. Adjustments should be made to surveillance and outbreak response strategies to prevent and reduce the effects of future outbreaks.
